# Unveiling the hidden threat: investigating gastrointestinal parasites and their costly impact on slaughtered livestock

**DOI:** 10.1590/S1984-29612024061

**Published:** 2024-10-07

**Authors:** Hafiz Muhammad Rizwan, Hafiz Muhammad Zohaib, Muhammad Sohail Sajid, Urfa Bin Tahir, Razia Kausar, Nadia Nazish, Mourad Ben Said, Nimra Anwar, Mahvish Maqbool, Dalia Fouad, Farid Shokry Ataya

**Affiliations:** 1 Section of Parasitology, Department of Pathobiology, KBCMA College of Veterinary and Animal Science, Narowal, Sub campus UVAS, Lahore, Pakistan; 2 Department of Parasitology, Faculty of Veterinary Science, University of Agriculture, Faisalabad, Pakistan; 3 IVF/Cell Culture Lab. U.S.-Pakistan Center for Advanced Studies in Agriculture and Food Security, University of Agriculture, Faisalabad, Pakistan; 4 Department of Anatomy, Faculty of Veterinary Science, University of Agriculture Faisalabad, Pakistan; 5 Department of Zoology, University of Sialkot, Pakistan; 6 Laboratory of Microbiology, National School of Veterinary Medicine of Sidi Thabet, University of Manouba, Manouba, Tunisia; 7 Department of Basic Sciences, Higher Institute of Biotechnology of Sidi Thabet, University of Manouba, Manouba, Tunisia; 8 Department of Pharmacology and Toxicology, Faculty of Bio-Sciences, University of Veterinary and Animal Sciences, Lahore, Pakistan; 9 Eastwood Lab, Department of Entomology, Virginia Tech University, Blacksburg, USA; 10 Department of Zoology, College of Science, King Saud University, Riyadh, Saudi Arabia; 11 Department of Biochemistry, College of Science, King Saud University, Riyadh, Saudi Arabia

**Keywords:** Parasitic infection, prevalence, organ condemnation, economic losses, ruminant faecal examination, postmortem examination, Infecção parasitária, prevalência, condenação de órgãos, perdas econômicas, exame fecal de ruminantes, exame post-mortem

## Abstract

This study investigated the prevalence of gastrointestinal (GI) parasites in ruminants slaughtered at the abattoir in district Narowal, Punjab, Pakistan. The overall prevalence of parasitic infection was determined to be 72.92% based on faecal examination. Among the ruminant species, goats exhibited a significantly higher (P < 0.05) prevalence of parasitic infection (78.63%) compared to cattle, buffalo, and sheep. Additionally, female ruminants showed a significantly higher (P<0.05) prevalence of infection (85.62%) compared to males (65.13%). The intestines (both small and large) of small and large ruminants were found to be significantly more affected, with a prevalence of 39.58% of parasitic infection compared to other examined organs. A total of ten parasitic genera were identified in ruminants, including hydatid cysts. Ruminants with a high burden of parasites (45.74%) significantly outnumbered those with light (23.40%) and moderate (30.85%) burdens. Economically, the estimated annual losses in Pakistan due to organ condemnation with GI parasites were substantial, amounting to Pak. Rs. 405.09/- million (USD = 1,428,760). These findings underscore the significance of GI parasite infections as a major animal health concern and a cause of significant economic losses in the research area.

## Resumo

Este estudo investigou a prevalência de parasitas gastrointestinais em ruminantes abatidos no matadouro do distrito de Narowal, Punjab, Paquistão. A prevalência geral de infecção parasitária foi determinada como sendo de 72,92% com base em exame fecal. Entre as espécies de ruminantes, os caprinos apresentaram uma prevalência significativamente maior (P < 0,05) de infecção parasitária (78,63%), em comparação com bovinos, búfalos e ovinos. Além disso, as fêmeas de ruminantes mostraram uma prevalência significativamente maior (P<0,05) de infecção (85,62%), comparando-se com os machos (65,13%). O intestino (tanto delgado quanto grosso) de pequenos e grandes ruminantes foi significativamente mais afetado, com uma prevalência de 39,58% de infecção parasitária em comparação com outros órgãos examinados. Um total de dez gêneros de parasitas foram identificados em ruminantes, incluindo cisto hidático. Ruminantes com uma alta carga de parasitas (45,74%) superaram, significativamente, aqueles com cargas leves (23,40%) e moderadas (30,85%). Economicamente, as perdas anuais estimadas no Paquistão, devido à condenação de órgãos com parasitas gastrointestinais, foram substanciais, totalizando 405,09 milhões de rupias paquistanesas (USD = 1.428.760). Esses resultados destacam a importância das infecções por parasitas gastrointestinais como uma grande preocupação com a saúde animal e causa de perdas econômicas significativas na área de pesquisa.

## Introduction

The livestock industry plays a vital role in the economies of developing nations, and Pakistan, being an agricultural country, heavily relies on this sector. However, the performance of the livestock sector has been consistently declining due to various factors, including social and political circumstances, climate conditions, and environmental concerns (Latif et al., 2010; Ahmad et al., 2017; Saleem et al., 2023). Among the key obstacles hampering the growth of commercial livestock industries are parasitic diseases. These diseases have a significant impact on the majority of agroecological zones and pose a serious threat to the global livestock industry (Vercruysse & Claerebout, 2001).

Parasitic infections lead to reduced efficiency and productivity of animals and increased mortality, directly affecting the income of farming communities (Batool et al., 2022). In young animals, mortality rates can soar above 40%, with each animal losing 6–13 kg of weight annually (Jittapalapong et al., 2011). Nematodes are identified as one of the most harmful and economically significant gastrointestinal parasites (GI) that infect ruminants, as supported by previous studies (Jurasek et al., 2010; Ahmad et al., 2017; Win et al., 2020). The susceptibility of animals to various GI parasites is attributed to factors such as unsanitary living conditions, inadequate treatment, close contact with pathogenic animals, and harsh climatic conditions (Gadahi et al., 2009; Dabasa et al., 2017). Environmental factors and the lack of awareness among animal owner’s further increase parasite infections (Tehmina et al., 2014).

Parasitic diseases pose a significant threat to the livestock industry in developing nations like Pakistan, hampering the growth and development of domestic livestock species. Among the various parasitic infections, helminthiasis severely impacts the majority of ruminants, resulting in anorexia, poor reproductive performance, weight loss, reduced disease resistance, and, in some cases, even death, leading to substantial economic losses (Ngategize et al., 1993). Studies have shown that gastrointestinal parasite diseases in large ruminants can cause up to a 16% loss in farmer profits and a 50% decrease in weight (Khan et al., 2022). Research conducted in Peshawar, Pakistan examined the incidence of different helminth infections in cattle, revealing a high prevalence of liver infections (77.5%), primarily caused by *Fasciola hepatica*, followed by *Paramphistomum* spp. and hydatid cysts. Hydatid cysts were also observed (2.5% recovered), while no signs of nematode infection were found in the lungs (Siddiqi & Shah, 1984).

The carcasses identified with parasitic diseases during postmortem inspections in abattoirs are either partially or entirely condemned. For livestock producers, such inspections are of paramount importance as they provide crucial knowledge to develop and implement effective animal health strategies for reducing and controlling parasite diseases (Jaja et al., 2017).

In Pakistan, comprehensive data on the prevalence and extent of gastrointestinal (GI) parasites, contamination of organs with GI parasites, and resulting economic losses in slaughtered ruminants are lacking. This study addresses these gaps by investigating the prevalence, impacts, and financial losses associated with GI parasite infections in slaughtered animals. Through pre-slaughter faecal examinations and postmortem investigations, the presence of internal parasite eggs and adult parasites in various organs and tissues was determined. Additionally, the economic impact of organ condemnation was evaluated.

## Material and Methods

### Study area and sample size determination

**Figure 1:**
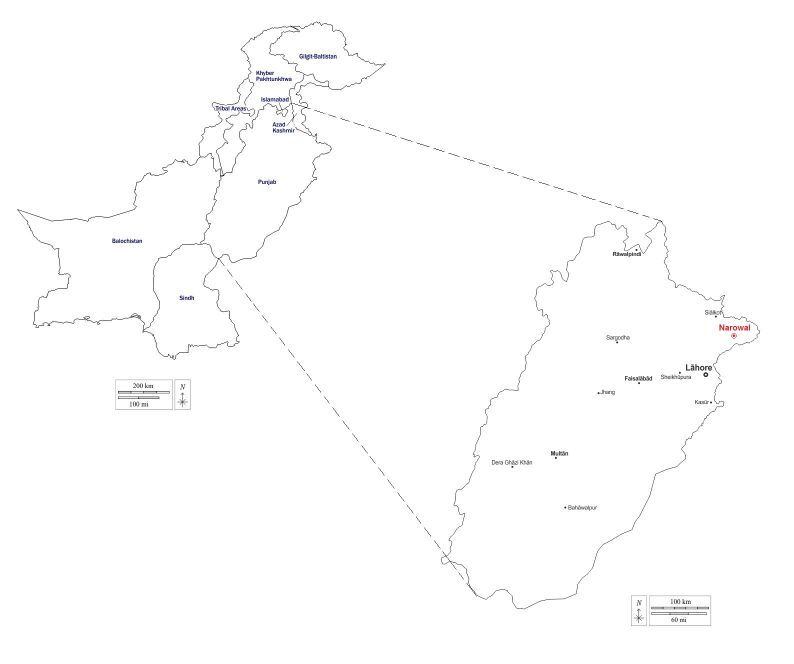
Map of Pakistan showing the studied Narowal district located in Punjab province.

The study was conducted in Narowal, a district situated in the northeastern region of Punjab province, Pakistan (Figure 1). Narowal district comprises three tehsils (subdivisions): Narowal, Shakargarh, and Zafarwal, encompassing a total of 74 Union Councils. To determine the appropriate sample size for the investigation, the formula provided by Thrusfield (2018) was employed, assuming an expected prevalence of 50%. By substituting the values into the formula, a sample size of 384 was calculated, ensuring a 95% confidence interval and 5% absolute precision.


$$n=\frac{1.96^{2}P_{\text{exp}}\;\left(1-P_{\text{exp}}\right)}{d^{2}}$$

Where, n = Sample size; *P_exp_ =* Expected prevalence; d^2^ = Desired precision

### Collection and examination of faecal samples

Each animal presented to the abattoir for slaughter was assigned a unique identification number, and relevant information such as sex, age, and species was recorded on the datasheet. The collection of faecal samples (n = 384) was conducted from January 2023 to June 2023 following the recommended protocols outlined by Soulsby (1982). Approximately 10-15 grams of faeces were obtained per rectum and placed in plastic vials containing a 3:1 formalin solution (3 parts formalin to 1-part sample). These collected samples were then transported to the Department of Pathobiology at the KBCMA College of Veterinary and Animal Sciences, Narowal, Sub-campus UVAS, Lahore, Pakistan, for further processing using standard procedures. The samples were stored in a freezer at 4°C until processing.

Identification of GI parasite eggs was carried out using conventional centrifugal flotation (Ahmad et al., 2017) and sedimentation (Adedokun et al., 2008) methods. For the centrifugal flotation method, approximately 3 grams of the faecal sample were mixed with a 12 mL flotation solution (saturated salt solution) to create a homogenous mixture. This mixture was poured through a strainer into a centrifuge tube and centrifuged at 1,200 RPM for 5 minutes. After centrifugation, the supernatant was carefully collected using a pipette and placed on a microscope slide for microscopic examination to identify parasite eggs. For the sedimentation method, approximately 3 grams of the faecal sample were mixed with water and filtered through a strainer into a centrifuge tube. The tube was then centrifuged at 1,500 RPM for 5 minutes to concentrate the eggs at the bottom. The supernatant was carefully decanted, and the sediment was resuspended in a small amount of water. A drop of the sediment was then placed on a microscope slide and examined under a microscope to identify parasite eggs.

Based on egg size (micrometry) and shape, the respective parasite genera were determined (Soulsby, 1982). To quantitatively examine the faeces (only for nematodes), the "Modified McMaster test" was employed to determine the egg content. The egg per gram (EPG) counts of faeces from the animals were then categorized as light, moderate, or heavy infestations. As per Soulsby (1982) and Urquhart et al. (1996), egg counts ranging from 100 to 600, 700 to 1000, and over 1000 per gram of faeces were classified as light, moderate, and heavy infections, respectively.

### Organ examination

To ascertain the presence of both mature and immature parastes, various organs including the liver, lungs, rumen, abomasum, and intestines (both small and large) of slaughtered animals were carefully collected and examined. A thorough examination was conducted on each organ to screen for adult and immature parasites. Specifically, the liver's bile ducts and any necrotic areas were quickly inspected for the presence of *Fasciola* spp., hydatid cysts, and *Paramphistomum* spp. Additionally, the lungs of the slaughtered animals were checked for the existence of hydatid cysts. The abomasum and rumen were opened and examined to identify nematodes and rumen flukes, respectively. Intestinal contents were squeezed out and examined for nematodes and cestodes. To preserve all the recovered worms for further investigation, 70% ethanol was utilized. The identification of parasites was carried out based on the gross and/or microscopic morphological characteristics described in Soulsby (1982).

### Determination of economic losses due to organ condemnation

For the assessment of economic losses, all organs affected by parasites were considered condemned. The organs were considered condemned when the veterinary doctor on duty examined them and recommended condemnation. This assessment was conducted according to the guidelines set by the Ministry of National Food Security & Research (Government of Pakistan) to determine the impact of parasitic infections on organ viability and public health safety. To calculate the annual loss resulting from organ condemnation, the total number of animals slaughtered in the abattoir each year and the average retail price of organs at the abattoir were taken into account. Information on the average market price of the organs was obtained through discussions with butchers and abattoir staff. The annual slaughter rate of the abattoir was determined using historical data from previous years available in the abattoir records. To calculate the annual financial loss incurred due to the complete condemnation of organs, the method described by Jaja et al. (2017) was employed, which involves multiplying the mean number of ruminants slaughtered, the mean cost of organs, and the prevalence of parasite infection. By implementing the above-mentioned approach, the study was able to quantify the economic impact caused by the condemnation of organs affected by parasitic infections.

### Statistical analyses

Descriptive data analyses were employed to elucidate the prevalence of parasites. The Chi-square test was utilized to assess variations in the prevalence of GI parasites among different independent factors, such as species, sex, and age groups, which were treated as categorical variables. Data analysis was performed using SPSS 17.0 software (SPSS Inc., Chicago, USA). Statistical significance of risk factors was determined based on the P-value, and factors were considered statistically significant if their P-value was less than 0.05. This allowed for the identification of key risk factors associated with parasite prevalence in the studied population.

## Results

### Prevalence of parasitic infection

**Figure 2:**
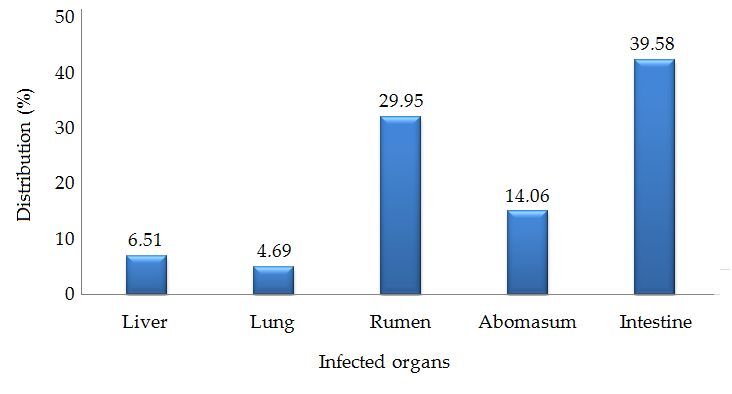
Distribution rates of parasitic infections according to infected organs of ruminants analyzed in this study.

The overall prevalence of parasitic infection in ruminants in district Narowal, Punjab, Pakistan, was found to be 72.92%. Among the ruminant species, the goat population exhibited a significantly higher (P < 0.025) prevalence of parasitic infection (78.63%) compared to cattle (74.77%), buffalo (76.56%), and sheep (60.87%). The prevalence of parasitic infection was significantly (P < 0.05) higher in female ruminants (85.62%) than in males (65.13%). However, there was no significant (P > 0.05) association observed between age or sampling months and the prevalence of parasites (Table 1). The intestines showed a significantly (P < 0.05) higher prevalence (39.58%) of parasitic infection compared to other examined organs (Figure 2).

**Table 1:** Prevalence rates of parasitic infections in ruminants of district Narowal, Punjab, Pakistan.

**Variable**	**Level**	**Examined**	**Positive**	**Prevalence (%±C.I.)**	***X*^2^-value**	**P-value**
Species	Cattle	111	83	74.77±0.080	9.322	0.025^*^
	Buffalo	64	49	76.56±0.103		
	Sheep	92	56	60.87±0.099		
	Goat	117	92	78.63±0.074		
Sex	Male	238	155	65.13±0.060	19.238	0.000^*^
	Female	146	125	85.62±0.056		
Age	Young	101	68	67.33±0.092	2.168	0.141
	Adult	283	212	74.91±0.050		
Months	January	64	45	70.31±0.111	5.96	0.310
	February	64	47	73.44±0.107		
	March	64	51	79.69±0.098		
	April	64	41	64.06±0.117		
	May	64	51	79.69±0.098		
	June	64	45	70.31±0.111		
Total		384	280	72.91±0.045		

Abbreviations: C.I.: 95% confidence interval;

* Statistically significant, p < 0.05.

In the sheep and goat populations, females showed a significantly higher prevalence of parasitic infection than males (P < 0.05). In sheep, adults had a significantly higher prevalence than young (P < 0.05). However, in goats, age showed no significant association with parasitic infection (P > 0.05). The prevalence of parasitic infection in female sheep was 90.90%, in males was 51.43%, in young sheep (lambs) was 44.44%, and in adults was 67.69%. In goats, the prevalence was 97.72% in females, 67.12% in males, 85.71% in young goats (kids), and 75.61% in adults. In cattle and buffalo, both age and sex showed no significant association with parasitic infection (P > 0.05). The prevalence of parasitic infection in female cattle was 75.93%, in males was 73.68%, in young cattle (calves) was 66.67%, and in adults was 77.78%. In buffalo, the prevalence was 80.77% in females, 68.69% in males, 60.00% in young buffalo (buffalo calves), and 75.44% in adults.

### Identified internal parasites

**Figure 3:**
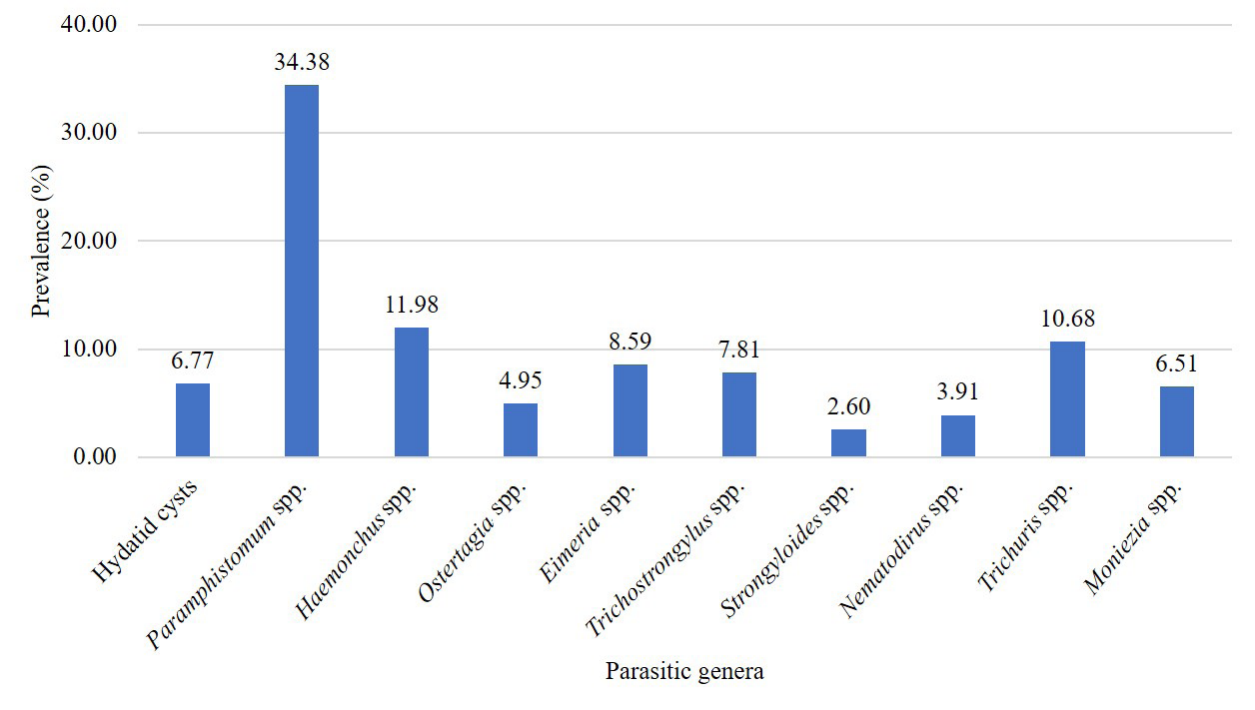
Prevalence rates of each parasite genera identified in the studied ruminant population of district Narowal, Punjab, Pakistan.

**Figure 4:**
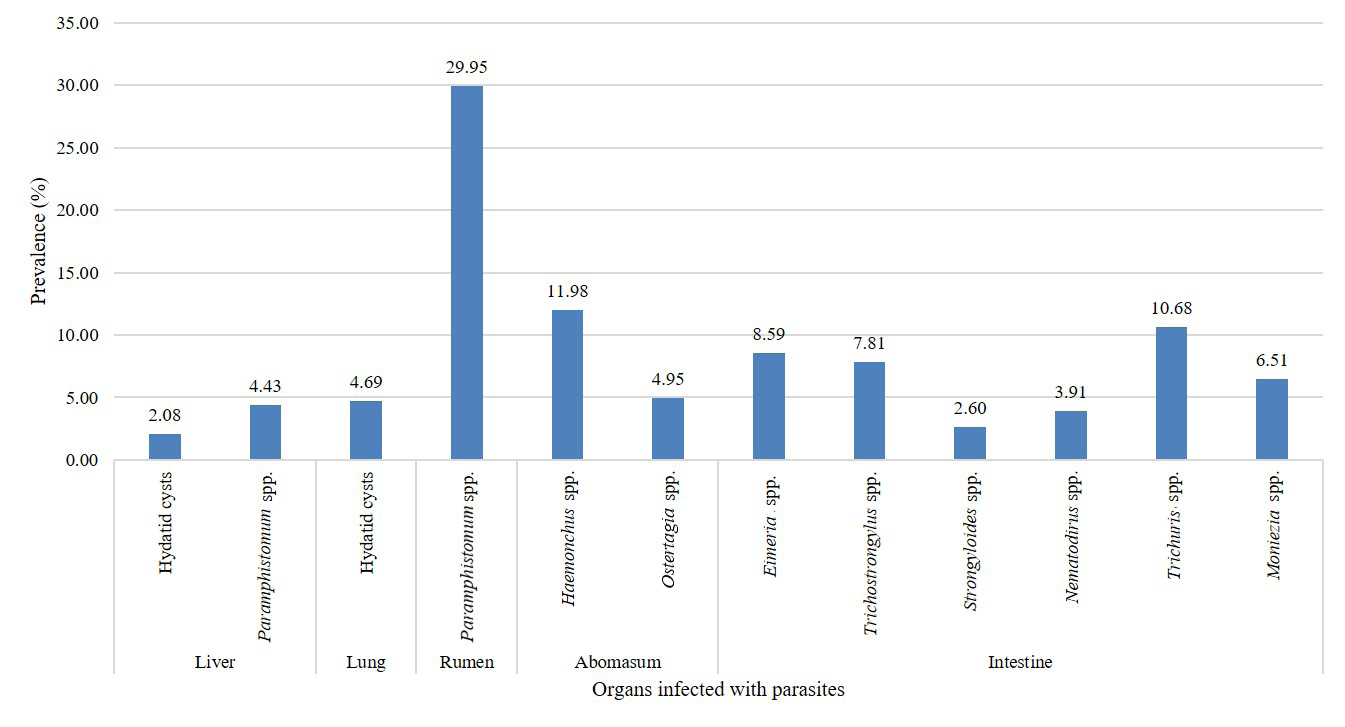
Prevalence of different genera of parasites identified from different organs.

A total of ten parasitic genera were identified in ruminants, comprising one intermediate form hydatid cysts (6.77%), as well as adults and eggs of nine genera: *Paramphistomum* spp. (34.38%), *Haemonchus* spp. (11.98%), *Trichuris* spp. (10.68%), *Eimeria* spp. (8.59%), *Trichostrongylus* spp. (7.81%), *Moniezia* spp. (6.51%), *Ostertagia* spp. (4.95%), *Nematodirus* spp. (3.91%), and *Strongyloides* spp. (2.6%) (Figure 3). The prevalence of hydatid cysts was higher in the lungs (4.69%) than in the liver (2.08%). Other than rumen, *Paramphistomum* spp. was also found in the liver. The prevalence of *Paramphistomum* spp. was significantly (P < 0.05) higher in the rumen (29.95%) compared to the liver (4.43%), and it was found to be significantly more prevalent than other parasitic genera. The prevalence of different parasites identified from different organs is given in Figure 4.

### Burden and prevalence of single of multiple parasitic infections

The number of animals with high parasitic load was significantly (P < 0.05) higher (45.74%) than the light (23.40%) and moderate (30.85%) burdens of GI parasites in ruminants (Table 2). The ruminant population in district Narowal was significantly (P < 0.05) more infected with one parasitic genus (50.78%) than with two (19.01%) or three (3.13%) parasitic genera (Table 3).

**Table 2:** Parasitic load (egg per gram) of parasites in ruminants of district Narowal, Punjab, Pakistan.

**Parasitic load**	**Number**	**Rate (%)**	***X*^2^-value**	**P-value**
Light	22	23.40	15.182	0.001^*^
Moderate	29	30.85		
High	43	45.74		

* Statistically significant, p < 0.05.

**Table 3:** Prevalence of single and multiple parasitic genera infections in ruminants of district Narowal, Punjab, Pakistan.

**Infection type**	**Number**	**Prevalence rate (%±C.I.** 1 **)**	***X*^2^-value**	**P-value**
One parasitic genus	195	50.78±0.050	255.79	0.000^*^
Two parasitic genera	73	19.01±0.039		
Three parasitic genera	12	3.13±0.017		

1 C.I.: 95% confidence interval;

* Statistically significant, p < 0.05.

### Economic losses due to organ condemnation

The estimated annual economic losses due to condemnation of organs (liver, lung, and rumen) with GI parasites amounted to Pak. Rs. 405.09/- million (USD = 1428,760). The rumen condemnation (Pak. Rs. 236.87/- million; USD = 835,445) accounted for significantly higher economic losses compared to the liver (Pak. Rs. 142.54/-million; USD = 502,741) and lungs (Pak. Rs. 25.68/-million; USD = 90,573) (Table 4).

**Table 4:** Total estimated annual economic losses due to condemnation of organs (liver, lung, and rumen) with gastrointestinal parasites.

**Organ**	**Animal**	**Mean number of ruminants slaughtered (a)**	**Mean cost (b)**	**Prevalence (c)**	**Annual loss = a × b × c**	
					**In Pak Rs.**	**In USD**
Liver	Large ruminants	6408	1800	10.29	118688976	418618
	Small ruminants	8900	800	3.35	23852000	84126
Lung	Large ruminants	6408	450	7.43	21425148	75566
	Small ruminants	8900	200	2.39	4254200	15004
Rumen	Large ruminants	6408	700	37.14	166595184	587584
	Small ruminants	8900	300	26.32	70274400	247859
Total					405089908	1428760

## Discussion

Regarding the overall prevalence of parasitic infection in Lorestan province, Iran, Ezatpour et al. (2014) reported a lower prevalence of GI parasites (23.3%) compared to the findings of the current study. On the other hand, in Minna modern abattoir, Niger state, Nigeria, prevalence (69.64%) is comparable to the prevalence observed in our study (Eke et al., 2019). In contrast, Bansal et al. (2015) reported a higher prevalence (90.05%) in India compared to our study. Numerous investigations have been conducted in Pakistan to assess the prevalence of GI parasites, with lower prevalence reported in Sialkot (32.6%) and Chakwal (58.13%) compared to our study (Rizwan et al., 2017; Abbas et al., 2021). The variations in parasitic prevalence may be attributed to differences in climatic conditions, agro-ecology of the study region, availability of veterinary services, management practices, grazing patterns, irrational use of anthelmintics, and the association of peasants (Dabasa et al., 2017).

The present study's findings align with previous research by Islam & Taimur (2008), Gadahi et al. (2009), and Yadav et al. (2006), indicating that the prevalence of GI parasite infections varies among different ruminant species. For instance, in Pakistan, buffaloes (63.55%) exhibited a higher prevalence of parasitic infection compared to cattle (55.61%) (Khan et al., 2022). In India, Choubisa & Jaroli (2013) reported the highest prevalence of parasite illnesses in cattle (93.84%), followed by goats (82.97%), sheep (55.42%), and buffaloes (46.29%). The variation in parasitism among different species can be attributed to their distinct grazing habits, gastrointestinal physiology, and genetic resistance to GI parasites (Islam & Taimur, 2008).

Similar to our study's findings, research conducted in Lower Dir, Khyber Pakhtunkhwa, Pakistan, revealed that female cows (62.58%) and female buffaloes (77.33%) were more frequently affected by GI parasite infections compared to males (Khan et al., 2022). Similarly, Abbas et al. (2021) reported a higher likelihood of GI parasite illnesses in female animals than in male animals. The susceptibility of animals to diseases can vary based on their sex, which may be influenced by hormonal control and genetic predisposition.

Based on research conducted in neighboring regions, the prevalence rates of parasite diseases in ruminants may be influenced by the species and age of the animals. For instance, a study conducted in the western region of Punjab, India, revealed that adult ruminants were more susceptible to parasite infection (nematode) compared to young ones (Singh et al., 2017). Ruminants older than 2 years showed a higher susceptibility to endo-parasitism, as also observed by Biu et al. (2009) and Uddin et al. (2006). These findings are consistent with Hassan et al. (2019) observation that age may be a risk factor, with older ruminants being more vulnerable to GI parasites than younger ones. Older animals showed higher susceptibility to endo-parasitism, likely due to increased exposure over time. Stress factors such as poor nutrition, concurrent diseases, and suboptimal living conditions can increase this susceptibility (Petersen et al., 1992).

Variations in the incidence of GI parasite infections were observed during different months and seasons. These findings differ from those of Maqbool et al. (2002), who reported a lower frequency during the summer in Pakistan (9.0%) and higher prevalence in spring (20.0%), followed by winter (13.0%). Similarly, Yadav et al. (2006) found a higher prevalence during the rainy season compared to other times of the year. In India, Singh et al. (2017) noted the highest prevalence of GI parasite infections in ruminants during the monsoon season (90.10%), followed by winter and summer (83.84%).

The prevalence of hydatid cysts was found to be 6.22% in a study conducted in three different slaughterhouses across the Pakistani provinces of Punjab, Khyber Pakhtunkhwa, and Azad Jammu and Kashmir, which is higher than the prevalence reported in the current study (Saleem et al., 2023). In a study by Khedri et al. (2021), liver contamination from parasites was higher at 7.5% compared to 3.6% for the lungs. Our findings were also higher than those of Gareh et al. (2021), who reported a prevalence of 16.66% of abomasum samples infected with *Haemonchus*. Additionally, Hassan et al. (2019) found that the omentum of the gut had a significant infection rate of 31.8% of *Taenia hydatigena* metacestode (*Cysticercus tenuicollis*).

In contrast to our findings, Molla et al. (2023) identified mild, moderate, and high worm egg infections in 82.6%, 12.9%, and 4.5% of the animals, respectively. Another study reported the lowest count of strongyle eggs at 136.39 EPG across all samples. Khan et al. (2022) documented mean EPG values of 143.30 for cattle and 122.56 for buffaloes. The variation in the parasite infection load may be attributed to differences in management practices, animal immunity, and the level of contamination in grazing areas. Understanding these factors is vital for developing effective strategies to control and mitigate the burden of GI helminths in ruminants.

Marskole et al. (2016) reported a higher prevalence of one parasitic genus infection compared to combined infections, which aligns with the findings of our study. Similarly, Ruhoollah et al. (2021) observed that one parasitic genus had a prevalence of 89.20%, two parasitic genera infection was at 48.36%, and three parasitic genera infection was found in 25.54% of cases, similar to our results. In contrast, Tiele et al. (2023) reported that around 40.75% of cattle had two or more parasitic genera, while only 26.5% of cattle had a single parasitic genus infection. The variation in infection rates may be attributed to differences in weather-related grazing behavior and other risk factors influencing the prevalence of parasitism in small ruminants, as previously discussed.

Regarding the economic impact of parasitic diseases, Latif et al. (2010) estimated the cost of cystic echinococcosis in Pakistan to be 26.5 million rupees annually. Ahmad et al. (2017) calculated direct and indirect economic losses caused by fasciolosis in the Sargodha area of Punjab, Pakistan, which amounted to USD 0.036 million and USD 0.177 million, respectively. In Iran, Khedri et al. (2021) projected overall losses due to parasite-related condemnation to be USD 3,191,879 over an eight-year period, with liver condemnation accounting for USD 2,937,727, lung condemnation for USD 78,502, and carcass condemnation for USD 175,650. Similarly, in Nigeria, Molla et al. (2023) evaluated the economic impact of parasitic diseases found at abattoirs and reported significant losses due to fasciolosis (USD 220,369.94), hydatidosis (USD 52,135.62), dicrocoeliosis (USD 10,238.50), cysticercosis (USD 2,221.23), and oesophagostomiasis (USD 19,168.53). It is worth noting that these estimates may not fully encompass all indirect losses, such as decreased production, veterinary care expenses, and animal fatalities, which would likely contribute to higher total financial losses.

One limitation of this study is that a total worm count was not conducted, primarily because butchers were often in a hurry to open their shops, which limited the time available for thorough examination and collection of all worms. Although we tried to examine and collect worms from each infected organ, this approach may not fully capture the worm burden. Additionally, the economic losses were calculated solely based on condemned organs and did not account for other significant factors such as reduced weight gain, medicinal costs, and decreased milk production. Future studies should aim to include adult worm abundance data and a more comprehensive evaluation of economic impacts to provide a fuller understanding of parasitic infections.

## Conclusions

This study sheds light on the prevalence of GI parasite infections in slaughtered ruminants in the Narowal district of Punjab, Pakistan, revealing a substantial burden of infection. Both single and combined GI parasite infections are common in the region, adversely affecting the meat industry with potential consequences like early deaths, reduced body weights, and decreased milk production. The estimated financial loss might be an underestimation as indirect costs are not considered. Implementing preventive and control measures is crucial to address parasitic burdens effectively. Regular examination of GI parasite prevalence in slaughterhouses, especially in underdeveloped areas, is vital to assess control program success. Faecal examination and postmortem surveys are valuable tools for monitoring and managing GI parasite infections. Proactive efforts to combat parasitic infections will enhance animal health, productivity, and overall economic efficiency in the livestock industry.
